# Maternity and Mothering

**Published:** 1911-12-30

**Authors:** 


### MATERNITY AND MOTHERING.

Inquiries and Correspondence are invited.

### AN AMERICAN SYSTEM OF INFANT FEEDING.

In spite of education, legislation (such as the maternity benefit of the Insurance Bill, conditional upon a month's abstention from work), and the persuasive eloquence of the medical profession, there is still a large percentage of babies who for one reason or another are not breast-fed as they should be; and, though this percentage will, it may be hoped, steadily decrease, it cannot ever be completely eliminated, and will not even be very greatly diminished within the next generation or so. Substitute feeding must necessarily, therefore, continue to demand the most minute attention in both principles and details; for the great advances made in the last thirty years in this subject have by no means exhausted room for further improvements.

PATENT MILK SUBSTITUTES.

For a decade or so, there has been among some schools of pediatrists a tendency towards complicated and expensive procedures for the preparation of substitute-milk suitable for the needs of infants at various stages of their development. Especially is this true of the United States, where some of the leading authorities, whose names carry weight all the world over, have adopted, practised, and taught "percentage methods," "milk prescriptions," and so on, of such complexity that only in very large cities are facilities to be found for carrying out their instructions.

In England these complicated methods have found favour with only a few of the specialists, though a good many have thought the underlying principle worthy of modification into a more practical and common-sense elaboration of details. The Vincent Square Infants' Hospital is perhaps the only institution where the percentage methods have been reverenced to the same extent as in America.

It is interesting to note that there are now some signs of a reversion to simpler milk modifications across the Atlantic. A month or two ago a New York specialist, Dr. R. R. Dennett, read a paper * in which he renounces the percentage feedings, cream and whey mixtures, cereal gruels, and other "fads," as he calls them; and confesses his humiliation in the discovery that milk, water, and sugar can be arranged to suit all healthy babies more successfully than the ingenious contrivances to which he has hitherto pinned his faith. In this reaction against over-elaboration he lays down certain rules which err perhaps a little on the side of rigidity: babies are not all ready-made to the same pattern, nor will a stereotyped régime suit them all, even in health.

However, the simplicity of the system is a great point in its favour, and those who have the responsibility of hand-feeding small infants may well put it to the test of experience.

THE RULE.

A child will thrive and gain weight, says Dr. Dennett, on a given quantity of (whole) milk and sugar, which amount is proportional to its weight. The milk must, of course, be diluted with water to suit the digestive system of the child; but the exact dilution does not affect the main principle, nor is it of the utmost importance. The food (milk and sugar, to wit) should be made as dilute as is practicable by adding sufficient water to bring the total amount taken in twenty-four hours up to the full capacity of the baby. That is to say, the infant requires per diem so many ounces of milk and so many ounces of sugar for each pound of its body-weight, and irrespective of its age.

This conception has, it must be admitted, the merit of simplicity; and every doctor knows that the weight of a child is a more important guide to its dietetic requirements than its precise age. But the factor of which insufficient account is taken is that of the milk; for the wide variations between the milk of different breeds, or of separate individuals of the same breed (and even of individual cows at different times of year, different ages, etc.) are well known to be frequent sources of confusion and alimentary disturbance in the children to whom substitute milks are offered. It is true that the author stipulates for a milk con-

* *American Journal of Obstetrics and Gynæcology*, November 1911, p. 694.

taining 4 per cent. of fat; but it is doubtful whether he will obtain therewith uniformity of the remaining constituents.

At all events, he gives a healthy baby, which has no digestive disturbance, twice as many ounces of milk in a day as it weighs in pounds; and of sugar (except in the case of those under one month of age) one ounce and a half of dextrose to the total quantity of food.

Thus the weight of sugar given does not vary with the weight of the child, whereas that of milk does so directly. The feeds are arranged by diluting with water to suit the age and digestive powers of the particular child. As a general rule each feed contains one ounce for each month of age, with one ounce over. This does not hold, naturally, under one month. It may be added that the author is not in favour of great dilutions, and that his mixtures are rather stronger than those generally advised for different-age periods.

The Exceptions.

There are four classes to whom these rules do not apply.

New-born babies are given two and a half ounces of milk with seven and a half ounces of water, divided into ten two-hourly feeds of one ounce each. Gradually more is given; thus at a week, all being well, one might order seven ounces of milk, thirteen ounces of water, and two teaspoonfuls of sugar, divided into ten feeds of two ounces each. From this time onwards the sugar is gradually increased up to one ounce in twenty-four hours; and the milk is increased up to ten ounces a day, with an equal quantity of water. This standard can, as a rule, be reached (according to Dr. Dennett) by the end of the third week; and on this diet the baby will gain in weight up to the end of the first month.

The next class of the exceptions to the general rule comprises fat infants over eight months of age. For these the rule is modified by subtracting from the total calculated quantity of milk per diem four or six ounces, or alternatively by decreasing the amount of sugar. In caloric value, one ounce of sugar is equivalent to six ounces of milk.

Atrophic or marasmic babies constitute another special class to whom the ordinary rules do not apply.

When once digestive disturbance has been got over, by feeding with very dilute mixtures or in any other way, the emaciation which remains often renders necessary an increase beyond the calculated daily requirement. Thus, if digestion will stand it, the sugar may be raised to two ounces a day, or three to five ounces of milk added beyond the quantity required by a normal baby. An example is given of a marasmic baby of four months, weighing eight and a half pounds, which would thrive upon nineteen ounces of milk, seventeen of water, and two of dextrose; or, alternatively, upon twenty-two ounces of milk, fifteen of water, and one and a half of sugar.

Digestive Disturbances.

Lastly, but most important of all, the case is considered of babies with gastric and intestinal troubles.

Here stress is laid upon the importance of omitting all sugar for the time being: this, together with a weak milk-and-water mixture, will often bring about a marked improvement within a few days. Since the milk mixture is unduly dilute, shorter intervals between the feeds are advised to compensate. When vomiting is a prominent feature of the case, reduction of the total quantity given at each feed is regarded as of greater importance than great dilution.

As an illustration, the author says that a baby getting ten ounces of milk and twenty ounces of water, three ounces every two hours, may vomit constantly. But if two ounces at a time are given of a mixture of ten ounces of milk and ten ounces of water, the vomiting will often cease at once. While agreement with the little-and-often principle for the diet of babies with digestive disturbance will be fairly general, it is not so easy to concede that the treatment of such disturbances is so simple and straightforward as Dr. Dennett represents it. Still, the main contentions of his paper, and his appeal for simplification, will not fail to obtain approbation in Great Britain; and are of interest in connection with the "milk prescription" and the reserve with which it has been adopted on this side of the Atlantic.

